# Expression and Significance of Immune Checkpoints in Clear Cell Carcinoma of the Uterine Cervix

**DOI:** 10.1155/2020/1283632

**Published:** 2020-04-03

**Authors:** Liju Zong, Qianqian Zhang, Yuncan Zhou, Yujia Kong, Shuangni Yu, Jie Chen, Youzhong Zhang, Yang Xiang

**Affiliations:** ^1^Department of Obstetrics and Gynaecology, Peking Union Medical College Hospital, Chinese Academy of Medical Sciences and Peking Union Medical College, Beijing 100730, China; ^2^Department of Pathology, Peking Union Medical College Hospital, Chinese Academy of Medical Sciences and Peking Union Medical College, Beijing 100730, China; ^3^Department of Obstetrics and Gynaecology, Qilu Hospital, Shandong University, Ji'nan 250012, China; ^4^Department of Obstetrics and Gynaecology, The Second Affiliated Hospital of Shandong First Medical University, Tai'an 271000, China

## Abstract

The purpose of this study was to investigate the expression levels of the immune checkpoint proteins, programmed cell death-ligand 1 (PD-L1), B7-H3, B7-H4, and V-domain Ig suppressor of T cell activation (VISTA), as well as the significance thereof, in clear cell carcinoma (CCC) of the cervix (a rare histological subtype of cervical cancer). We also compared the expression statuses of these biomarkers in cervical CCCs with those in cervical squamous cell carcinomas (SCCs). We evaluated the expression of PD-L1, B7-H3, B7-H4, and VISTA in 50 cervical CCCs and 100 SCCs using immunohistochemical staining and investigated the associations between these markers, clinicopathologic features, and survival in patients with CCCs. Of the cervical CCC samples examined, 22%, 16%, 32%, and 34% were positive for PD-L1, B7-H3, B7-H4, and VISTA, respectively. Nineteen samples (38%) were negative for all 4 of these markers, whereas 31 (62%) expressed at least 1 marker. None of these markers was associated with the investigated clinicopathologic variables or patient survival. PD-L1, B7-H3, and VISTA were observed significantly more frequently in SCCs than in CCCs of the cervix. Our study confirmed the expression of immune checkpoint proteins in cervical CCCs and indicated their nonredundant and complementary roles. As such, our data suggest that monotherapeutic immune checkpoint blockade may not be sufficiently effective in patients with cervical CCC.

## 1. Introduction

Clear cell carcinoma (CCC) of the uterine cervix is a rare histological subtype of cervical cancer that accounts for 4% of all cervical adenocarcinomas globally [1]. It has been reported to be associated with intrauterine exposure to diethylstilboestrol (DES); however, patients with CCC of the cervix who have no history of intrauterine DES exposure have recently been reported [2]. In contrast to squamous cell carcinoma (SCC) or other subtypes of adenocarcinoma, cervical CCC is aetiologically unrelated to human papillomavirus (HPV) infection [3–5] and is classified as a non-HPV-associated adenocarcinoma in the newly proposed International Endocervical Adenocarcinoma Criteria and Classification (IECC) [5, 6]. Owing to the rarity of this disease, its aetiology remains unknown, and its associated cancer immune microenvironment has not been well characterized.

Immune checkpoints such as programmed cell death 1 (PD-1) and its ligand (PD-L1) play essential roles in antitumour immunity, and their blockade has been shown to improve outcomes in patients with several types of malignancies [7]. The PD-1 inhibitor pembrolizumab has been approved for the treatment of patients with recurrent or metastatic cervical cancer expressing PD-L1. While the expression and clinical significance of the immune checkpoints PD-L1, B7-H3, and B7-H4 have been reported in common types of cervical cancer [8–11], the immune microenvironment and expression status of immune checkpoints in CCC of the cervix remain unknown.

The objectives of this study were to assess the expression of PD-L1, B7-H3, B7-H4, and the novel immune checkpoint V-domain Ig suppressor of T cell activation (VISTA); to investigate the associations between the expression of these markers, clinicopathological features, and patient survival; to compare the immune microenvironment of CCC with that of cervical SCC; and to better understand the role of immune checkpoints in the tumour immunology of cervical CCC.

## 2. Materials and Methods

### 2.1. Patients

This retrospective study included 50 patients with International Federation of Gynaecology and Obstetrics (FIGO) 2009 stage I–II primary CCC of the cervix who underwent surgical resections between January 2005 and December 2017 at Qilu Hospital of Shandong University (Jinan, China) and Peking Union Medical College Hospital (Beijing, China); all patients had adequate tumour samples for immunohistochemistry. Patients with mixed CCC and endocervical adenocarcinoma were excluded from the study. Clinicopathologic characteristics including age at diagnosis, tumour size, depth of stromal invasion, parametrial involvement, lymphovascular space invasion (LVSI), number of metastatic lymph nodes, treatment modality, and follow-up were recorded. To compare immune checkpoint expression statuses in cervical CCC with those in cervical SCC, 100 cervical SCC samples from patients treated between January 2012 and December 2013 were immunohistochemically stained for immune checkpoint proteins. The clinicopathologic parameters and survival information were described in accordance with the guidelines of the Reporting Recommendations for Tumour Marker Prognostic Studies [12]. This study was approved by the Institutional Review Board (SK-888); informed consent was not required owing to the retrospective nature of the study.

### 2.2. Morphologic Assessment

To determine a tumour's HPV status, two pathologists reviewed all haematoxylin and eosin-stained slides that contained tumour tissue according to the IECC system as described in the original study [6]. Briefly, tumours were classified as HPV-associated adenocarcinoma if HPV-associated features (i.e., easily identifiable apical mitotic figures and apoptotic bodies at scanning magnifications of ×20 or ×40) were present. Tumours were classified as non-HPV-associated if these features were not easily identifiable, visible only on high power, or not visible at all.

### 2.3. Immunohistochemistry and Evaluation

Immunohistochemistry (IHC) was performed using our laboratory protocol as described previously [13, 14]. Briefly, 4 *μ*m series of whole tissue sections were deparaffinized and subjected to heat-induced epitope retrieval with 10 mM sodium citrate (pH 6.0) at 95°C for 20 minutes. The endogenous peroxidase activity was quenched using a 0.3% hydrogen peroxide solution. The sections were incubated with primary antibodies against PD-L1, B7-H3, B7-H4, and VISTA. Human placentas were used as positive controls, whereas tissues that were not exposed to primary antibodies were used as negative controls.

For PD-L1 evaluation, we used the combined positive score (CPS), which was calculated as the number of PD-L1-stained cells (i.e., tumour cells (TCs), lymphocytes, and macrophages) divided by the total number of viable TCs and then multiplied by 100. CPS values < 1 were considered PD-L1-negative while those ≥1 were considered PD-L1-positive. Samples were considered positive for B7-H3 and B7-H4 when ≥5% of the TCs expressing these proteins at any intensity; the 5% cut-off was chosen based on a previous publication [15]. As VISTA expression on TCs and tumour-associated immune cells (TAICs) may have distinct prognostic values [16–18], we evaluated the expression of VISTA on these cell types separately. TCs and TAICS were each considered VISTA-positive when ≥5% of them were stained for this protein, as described in previous publications [17, 18].

### 2.4. Statistical Analysis

The chi-squared or Yates's chi-squared test was used to determine the association between categorical variables. Disease-free survival (DFS) was defined as the time between the date of surgery and that of recurrence, censoring, or the last follow-up; overall survival (OS) was defined as the time between the date of surgery and that of death from any cause, censoring, or the last follow-up. To identify predictors of prognosis, univariate survival analyses were performed using the Cox proportional hazards regression model, and hazard ratios (HRs) with 95% confidence intervals (95% CIs) for recurrence and death were calculated. All statistical analyses were conducted using the Statistical Package for the Social Sciences for Windows (version 20.0; IBM Corp., Armonk, NY, USA). A 2-sided *P* value < 0.05 was considered statistically significant.

## 3. Results

### 3.1. Clinicopathologic Features and Survival

The median age of the 50 patients was 40.5 years (range, 14–67 years); none had a history of exposure to DES. Eight patients were 25 years of age or younger, among whom 5 from the Peking Union Medical College Hospital cohort have been reported previously [19]. Two patients had congenital malformations of the lower genital tract and were described in our previous report [20]. Most patients (72%) were diagnosed with stage I disease. Thirty-three women (66%) had tumours with sizes <4 cm, and 21 patient samples (42%) presented with deep invasion of the stroma (≥1/2). LVSI was present in 6 patient samples, and 5 patients had metastatic lymph nodes. All patients underwent surgery (radical hysterectomy, cervical trachelectomy, or conization). Moreover, 10 patients received neoadjuvant chemotherapy before surgery, and most (92%) received postoperative adjuvant treatment. The clinicopathological features of these patients are summarized in Table 1. The tumours of all 50 patients were classified as non-HPV-associated adenocarcinomas. After a median follow-up of 51 months (range, 10–183 months), 7 patients (14%) developed recurrent disease and died of cervical cancer. The 5-year DFS and OS rates were 83.3% and 80.6%, respectively.

### 3.2. Expression of PD-L1, B7-H3, B7-H4, and VISTA in CCC of the Cervix

**Figure 1:**
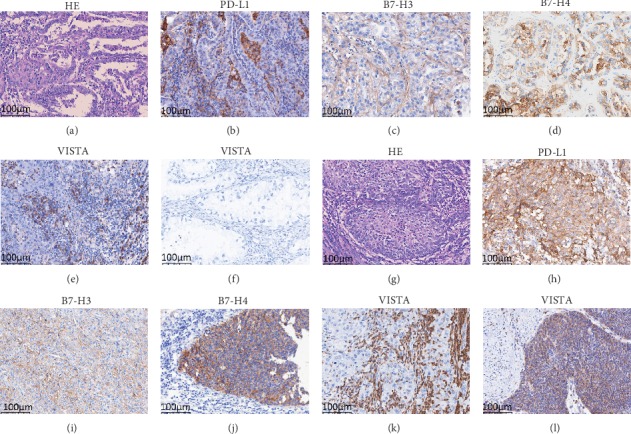
Haematoxylin and eosin (HE) staining as well as immune checkpoint protein expression in samples of clear cell carcinoma (a–f) and squamous cell carcinoma (g–l) of the uterine cervix: (a) HE, (b) positive programmed cell death-ligand 1 (PD-L1) expression in tumour cells (TCs) and tumour-associated immune cells (TAICs), (c) positive B7-H3 expression in TCs, (d) positive B7-H4 expression in TCs, (e) positive V-domain Ig suppressor of T cell activation (VISTA) expression in TAICs, (f) negative VISTA expression in TCs, (g) HE, (h) positive PD-L1 expression in TCs and TAICs, (i) positive B7-H3 expression in TCs, (j) positive B7-H4 expression in TCs, (k) positive VISTA expression in TAICs, and (l) positive VISTA in TCs. All the images are at ×200 magnification.

Consistent with our previous study [13, 14], the patterns of PD-L1, B7-H3, B7-H4, and VISTA staining in cervical CCC were predominantly membranous; representative images are shown Figure 1. PD-L1 was expressed in both TCs and TAICs and was positive in 11 samples (22%). Eight samples (16%) were B7-H3-positive while 16 (32%) were B7-H4-positive. VISTA-positive TAICs were typically observed at the invasive margin of the tumour or in the tumour-associated stroma. Seventeen samples (34%) showed VISTA expression in TAICs, while none showed its expression on TCs. Among the 39 PD-L1-negative samples, 20 (51%) were positive for B7-H3, B7-H4, or VISTA. Overall, 19 samples (38%) were negative for all 4 immune checkpoint proteins tested in this study, whereas 31 (62%) expressed at least 1 marker. Moreover, VISTA-positive samples were observed more frequently in PD-L1-positive samples than in PD-L1-negative counterparts (*P* = 0.001). Similarly, B7-H3-positive samples were more common in B7-H4-positive samples (*P* = 0.015).  Table 2 shows the association between PD-L1, B7-H3, B7-H4, and VISTA in CCC of the cervix.

### 3.3. Associations between Immune Checkpoint Protein Expression, Clinicopathologic Features, and Survival

We observed no association between the expression statuses of PD-L1, B7-H3, B7-H4, and VISTA on one hand and disease stage, tumour size, lymph node metastasis, depth of stromal invasion, or presence of LVSI on the other. As mentioned above, all tumours were non-HPV-associated adenocarcinomas, and no association was observed between HPV status and immune checkpoint expression. Univariate analysis revealed that a tumour size ≥ 4 cm (HR = 5.67, 95% CI, 1.09–29.55; *P* = 0.039) was significantly associated with a shorter OS, whereas neither the expression statuses of the 4 proteins nor the presence of other clinicopathologic features was associated with survival (Table 3).

### 3.4. Comparison of Markers in CCC versus SCC of the Cervix

B7-H3 was positive in a significantly higher proportion of patients with SCC (69%) than in those with CCC (16%; *P* < 0.001). Furthermore, PD-L1 was detected in a significantly higher proportion of patients with SCC (91%) than in those with CCC (22%; *P* < 0.001). Notably, 23% of patients with SCC showed VISTA expression on TCs, while none of those with CCC showed such expression. VISTA-positive TAICs were observed significantly more frequently in SCC than in CCC of the cervix (74% vs. 34%, *P* < 0.001). Lastly, no difference in B7-H4 expression was observed between SCCs and CCCs. Representative images of these markers in cervical SCC are shown in Figure 1, while comparisons of marker expression statuses in cervical CCCs versus SCCs are shown in Table 4.

## 4. Discussion

The B7 family of immune checkpoints (PD-L1, PD-L2, B7-H3, B7-H4, and VISTA) suppress T cell function and play important roles in tumour immune evasion [21]. The blockade of immune checkpoints has achieved remarkable clinical success in treating human malignancies, and immunotherapy targeting novel checkpoints such as VISTA has attracted growing interest. To our knowledge, ours is the first investigation of the B7 family of immune checkpoints to be performed in a sizable collection of CCCs of the cervix. Among the tumour samples that we investigated, we observed the expression of B7-H3 and B7-H4 in TCs, PD-L1 in both TCs and TAICs, and VISTA exclusively in TAICs. Although the positivity rate of any one of these immune checkpoints was relatively low, we found that 62% of tumour samples expressed at least 1 of these 4 immune checkpoint proteins and that half of PD-L1-negative samples were positive for at least 1 of the other 3 immune checkpoints. Moreover, we found that the distribution of these 4 proteins in cervical CCC was different from that in cervical SCC. Taken together, our data suggest that the B7 family of immune checkpoints play nonredundant and somewhat complementary roles in the immune evasion of cervical CCC. Thus, immune checkpoint inhibition as a monotherapy may not be adequately effective, while the combined blockade of at least 2 immune checkpoints may represent a novel therapeutic approach for CCC of the cervix.

The morphologic features of cervical CCC are similar to those of the ovary and uterine endometrium, comprising eosinophilic and hobnail cells with clear cytoplasm arranged in tubulocystic, papillary, or solid patterns. PD-L1 is reportedly expressed in CCC of the ovary and uterine endometrium [22, 23]. Willis et al. found that PD-L1 was present in 74% (17/23) and 76% (16/21) of ovarian and endometrial CCCs, respectively [22]. Moreover, Alldredge et al. found that PD-L1 was positive in 34.3% (12/35) and 60% (6/10) of ovarian and endometrial CCCs, respectively [23]. In the present study, PD-L1 was positive in 22% (11/50) of the cervical CCC samples. The differences in PD-L1 expression among gynaecologic CCCs may be attributed to the varying tumour microenvironments that have distinctive immune milieus and ought to be taken into consideration in future studies of PD-1/PD-L1 inhibitors as they warrant further investigation.

The expression of PD-L1 in cervical SCC and adenocarcinoma has previously been reported [10, 11]. Enwere et al. found that PD-L1 was positive in 88% of their patient samples that comprised 106 SCCs and 14 adenocarcinomas [11]; moreover, Heeren et al. reported that PD-L1 was positive in 54% (83/154) of the SCCs they investigated, which was a significantly higher proportion than its positivity rate in adenocarcinomas (14%; 7/49) [10]. Consistent with these results, PD-L1 positivity was observed more frequently in SCCs than in CCCs (91% vs. 22%) in the present study. The majority of cervical SCCs and common adenocarcinomas are associated with persistent high-risk HPV infection; however, CCC is aetiologically unrelated to HPV infection [3–5, 24, 25]. Interestingly, recent studies found that HPV-positive head and neck squamous cell carcinoma (HNSCC) and oropharyngeal squamous cell cancer were associated with positive PD-L1 expression [26, 27]. Moreover, Baruah et al. found that fibroblasts cocultured with HPV-positive (but not HPV-negative) HNSCC cells have upregulated PD-L1 expression in a TLR9-dependent fashion [28]. As all CCC samples were non-HPV-associated adenocarcinomas and no association was observed between HPV status and PD-L1 expression, the mechanisms of PD-L1 regulation in CCC require further exploration in future studies.

Early phase clinical trials targeting the novel immune checkpoints B7-H3, B7-H4, and VISTA alone or in combination with PD-1 inhibitors are ongoing. In the present study, we found that at least 1 of B7-H3, B7-H4, and VISTA was positive in half of the PD-L1-negative samples, suggesting a complementary role for these proteins in immune evasion. Consistent with our findings in CCCs of the cervix, Carvajal-Hausdorf et al. found that B7-H3 and PD-L1 were coexpressed in only 18% of patients with small-cell lung cancer, whereas no coexpression of B7-H4 or PD-L1 was observed [29]. These data suggest that the immune checkpoint proteins play nonredundant biological roles in cancer immunology. Moreover, Liu et al. demonstrated that the role of VISTA was nonredundant with the PD-1/PD-L1 pathway in terms of controlling T cell activation and that the combinatorial blockade of VISTA and PD-L1 achieved optimal tumour-eradicating therapeutic efficacy in colon cancer models. Taken together, combinatorial blockade of the B7 family of immune checkpoints may represent a novel therapeutic approach and achieve optimal therapeutic efficacy in human cancers, including CCC of the cervix.

The strengths of our study were the relatively comprehensive evaluation of the B7 family of immune checkpoints and a relatively large sample size (*N* = 50) from 2 tertiary hospitals. However, our study also had limitations. First, it was retrospective in nature and was therefore subject to inherent unavoidable biases. Second, we included only patients with FIGO stage I–II tumours, as samples were not available from patients with metastatic disease. Finally, it was difficult to analyse the coexpression of these immune checkpoints using immunohistochemistry; as such, multiplexed quantitative immunofluorescence would be required to objectively measure the expression of immune checkpoints within the tumour immune microenvironment. Despite these limitations, our pilot study that profiled the expression of candidate immunotherapy targets suggested that monotherapy involving immune checkpoint blockade is not sufficiently effective for patients with cervical CCC.

## 5. Conclusions

Our evaluation of the expression of 4 different immune checkpoint proteins in human CCC of the cervix revealed that, in contrast to cervical SCC, only a single immune checkpoint was expressed in a subset of CCCs; however, these expressed proteins varied across different CCCs. Our data indicate that immune checkpoints have nonredundant and complementary roles; hence, monotherapeutic immune checkpoint blockade may not be as effective for patients with cervical CCC as a combined approach.

## Figures and Tables

**Table 1:** Clinicopathological characteristics of patients with clear cell carcinoma of the uterine cervix (*N* = 50).

Variables	*N*	(%)
Age		
<40 years	25	50
≥40 years	25	50
FIGO 2009 stage		
IA	2	4
IB	34	68
IIA	12	24
IIB	2	4
Tumour size		
<4 cm	33	66
≥4 cm	17	34
Parametrial involvement		
Yes	4	8
No	46	92
LVSI		
Yes	6	12
No	44	88
Depth of stromal invasion		
<1/2	29	58
≥1/2	21	42
Lymph node metastasis		
No	45	90
Yes	5	10
Neoadjuvant therapy		
Yes	10	20
No	40	80
Adjuvant therapy		
Chemotherapy	18	36
Radiotherapy	6	12
Chemoradiotherapy	22	44
None	4	8

FIGO: International Federation of Gynaecology and Obstetrics; LVSI: lymphovascular space invasion.

**Table 2:** Association between PD-L1, B7-H3, B7-H4, and VISTA in clear cell carcinoma of theuterine cervix.

Variables	B7-H3		B7-H4		PD-L1	
	Negative	Positive	Negative	Positive	Negative	Positive
B7-H4	*P* = 0.015					
Negative	32	2				
Positive	10	6				
PD-L1	*P* = 0.809		*P* = 0.455			
Negative	32	7	25	14		
Positive	10	1	9	2		
VISTA	*P* = 0.320		*P* = 0.357		*P* = 0.001	
Negative	26	7	21	12	31	2
Positive	16	1	13	4	8	9

PD-L1: programmed cell death-ligand 1; VISTA: V-domain Ig suppressor of T cell activation.

**Table 3:** Univariate Cox regression analysis of survival among patients with clear cell carcinoma of the uterine cervix (*N* = 50).

Variables	Disease-free survival			Overall survival		
	HR	95% CI	*P* value	HR	95% CI	*P* value
Age						
<40 years	1.00			1.00		
≥40 years	0.67	0.15–2.98	0.596	0.67	0.15–3.00	0.601
Stage						
I	1.00			1.00		
II	2.81	0.34–23.40	0.340	3.01	0.36–25.10	0.308
Tumour size						
<4 cm	1.00			1.00		
≥4 cm	4.62	0.89–23.95	0.068	5.67	1.09–29.55	0.039
Parametrial involvement						
No	1.00			1.00		
Yes	3.88	0.75–20.05	0.106	3.80	0.74–19.64	0.111
LVSI						
No	1.00			1.00		
Yes	1.20	0.14–9.95	0.869	1.10	0.13–9.17	0.929
Depth of stromal invasion						
<1/2	1.00			1.00		
≥1/2	0.41	0.08–2.10	0.281	0.42	0.08–2.16	0.299
Lymph node metastasis						
No	1.00			1.00		
Yes	0.04	0.00–948.3	0.533	0.04	0.00–1492	0.553
B7-H3						
Negative	1.00			1.00		
Positive	0.04	0.00–179.5	0.447	0.04	0.00–138.9	0.431
B7-H4						
Negative	1.00			1.00		
Positive	0.35	0.04–2.88	0.326	0.38	0.05–3.20	0.376
PD-L1						
Negative	1.00			1.00		
Positive	1.40	0.27–7.20	0.691	1.25	0.24–6.44	0.792
VISTA						
Negative	1.00			1.00		
Positive	1.23	0.24–6.38	0.802	0.82	0.16–4.23	0.812

LVSI: lymphovascular space invasion; PD-L1: programmed cell death-ligand 1; VISTA: V-domain Ig suppressor of T cell activation; HR: hazard ratio; CI: confidence interval.

**Table 4:** Expression of PD-L1, B7-H3, B7-H4, and VISTA in clear cell carcinoma and squamous cell carcinoma of the uterine cervix.

Variables	CCC (*N* = 50)	SCC (*N* = 100)	*P* value
	*N*	*N*	
B7-H3			<0.001
Positive	8	69	
Negative	42	31	
B7-H4			0.612
Positive	16	28	
Negative	34	72	
PD-L1			<0.001
Positive	11	91	
Negative	39	9	
VISTA in TCs			<0.001
Positive	0	23	
Negative	50	77	
VISTA in TAICs			<0.001
Positive	17	74	
Negative	33	26	

PD-L1: programmed cell death-ligand 1; VISTA: V-domain Ig suppressor of T cell activation; CCC: clear cell carcinoma; SCC: squamous cell carcinoma; TCs: tumour cells; TAICs: tumour-associated immune cells.

## Data Availability

The data used to support the findings of this study are included within the article.
